# Betacellulin Induces Increased Retinal Vascular Permeability in Mice

**DOI:** 10.1371/journal.pone.0013444

**Published:** 2010-10-18

**Authors:** Bela Anand-Apte, Quteba Ebrahem, Alecia Cutler, Eric Farage, Masahiko Sugimoto, Joe Hollyfield, Judah Folkman

**Affiliations:** 1 Department of Ophthalmology, Cole Eye Institute, Cleveland Clinic Lerner College of Medicine, Cleveland, Ohio, United States of America; 2 Department of Molecular Medicine, Lerner Research Institute, Cleveland Clinic Lerner College of Medicine, Cleveland, Ohio, United States of America; 3 Vascular Biology, Children's Hospital, Boston, Massachusetts, United States of America; Leiden University Medical Center, Netherlands

## Abstract

**Background:**

Diabetic maculopathy, the leading cause of vision loss in patients with type 2 diabetes, is characterized by hyper-permeability of retinal blood vessels with subsequent formation of macular edema and hard exudates. The degree of hyperglycemia and duration of diabetes have been suggested to be good predictors of retinal complications. Intervention studies have determined that while intensive treatment of diabetes reduced the development of proliferative diabetic retinopathy it was associated with a two to three-fold increased risk of severe hypoglycemia. Thus we hypothesized the need to identify downstream glycemic targets, which induce retinal vascular permeability that could be targeted therapeutically without the additional risks associated with intensive treatment of the hyperglycemia. Betacellulin is a 32 kD member of the epidermal growth factor family with mitogenic properties for the retinal pigment epithelial cells. This led us to hypothesize a role for betacellulin in the retinal vascular complications associated with diabetes.

**Methods and Findings:**

In this study, using a mouse model of diabetes, we demonstrate that diabetic mice have accentuated retinal vascular permeability with a concomitant increased expression of a cleaved soluble form of betacellulin (s-Btc) in the retina. Intravitreal injection of soluble betacellulin induced retinal vascular permeability in normoglycemic and hyperglycemic mice. Western blot analysis of retinas from patients with diabetic retinopathy showed an increase in the active soluble form of betacellulin. In addition, an increase in the levels of A disintegrin and metalloproteinase (ADAM)-10 which plays a role in the cleavage of betacellulin was seen in the retinas of diabetic mice and humans.

**Conclusions:**

These results suggest that excessive amounts of betacellulin in the retina may contribute to the pathogenesis of diabetic macular edema.

## Introduction

The incidence of diabetes world-wide, is expected to reach epidemic proportions by 2025. Progression of diabetic retinopathy often results in diabetic macular edema, which is a consequence of the breakdown of the blood-retinal barrier, increased retinal vascular permeability and leakage of plasma from small blood vessels in the macula leading to loss of central vision. While epidemiological studies have suggested that glycemic control plays a major role in the development of vascular complications of diabetes[1], current insulin therapies for control of glucose metabolism have not been successful in the prevention of long-term complications[2], [3]. Intervention studies have determined that while intensive treatment of diabetes reduced the development of proliferative diabetic retinopathy [4], it was associated with a two to three-fold increased risk of severe hypoglycemia[5] as well as an increased risk of mortality from cardiovascular disease [6]. Laser photocoagulation as well as anti-VEGF therapies have shown significant promise in the treatment of proliferating vessels in proliferative diabetic retinopathy. However, DME appears to be more resistant to these treatment approaches, suggesting that other factors might contribute to this phenotype. These observations led us to hypothesize the presence of a novel factor that contributes to the increased retinal vascular permeability and/or retinopathy seen in diabetes.

Betacellulin (BTC) is a 32 kD member of the epidermal growth factor (EGF) family that was originally isolated from the conditioned medium of a mouse pancreatic β-cell tumor line[7]. BTC is a glycosylated (N- and O-linked) protein with an apparent molecular weight of 32 kDa, generated by cleavage of a 178-amino acid membrane anchored precursor protein pro-BTC. BTC binds and activates the EGF receptor (EGFR/erbB-1) and erbB-4[8], [9], [10]. BTC has been shown to be expressed in the human pancreas by pancreatic β cells [11], [12], [13] as well in other organs[7], induce differentiation of a pancreatic exocrine cell line into insulin secreting cells[13], [14], [15]and induce pancreatic β-cell neogenesis in diabetic mice[16]. Because of the proliferative effects of BTC on retinal pigment epithelial cells we hypothesized that it may contribute to increased retinal vascular permeability and the pathogenesis of diabetic macular edema. We have compelling evidence that betacellulin can increase retinal vascular permeability and may play a role in the pathogenesis of retinal vascular leakage in diabetes.

## Materials and Methods

### Materials

Recombinant human VEGF was a kind gift from Genentech, CA and recombinant betacellulin was purchased from R&D. Antibodies: polyclonal anti-mouse and anti-human betacellulin antibody (R&D), monoclonal anti-ADAM-10 (Calbiochem) and HRP-conjugated anti-rabbit, anti-mouse IgG antibodies (Amersham Pharmacia Biotech) and anti-goat IgG (Santa Cruz). Post mortem human eyes were obtained from he National Disease Research Interchange (NDRI), Philadelphia. Postmortem time ranged from 3–10 hours.

### Immunoblotting

Lysates from mouse or human retina prepared using sonication on ice with RIPA buffer (Boston Bioproducts) containing EDTA free protease inhibitors (Roche) were subjected to SDS-PAGE. Proteins were probed with antibody and detected with either a HRP-conjugated anti-rabbit or anti mouse IgG antibody (Amersham Pharmacia Biotech, Piscataway, NJ) followed by ECL. The blots were restripped with Restore™ solution (Thermo Scientific) for 30 minutes and reprobed as indicated.

### Animals

6–8 week old C57BL6 mice (Jackson Laboratories, Maine) were used in this study. The animals were cared for in accordance with the ARVO statement for the Use of Animals in Ophthalmic and Vision research. All experimental procedures used aseptic sterile techniques and were approved by the Animal Care and Use Committee of the Cleveland Clinic (ARC08792).

### Experimental Diabetes

6–8 week old mice were rendered diabetic with 3 consecutive daily intraperitoneal injections of STZ (55 mg/Kg) freshly dissolved in citrate buffer (pH 4.5). Control mice were given injections of buffer alone. Development of diabetes (defined by blood glucose greater than 250 mg/dl) was confirmed 1 week after the initial injection (One Touch®ltra®Test Strips and One Touch®UltraMini™glucometer).

### Blood retinal barrier quantitation

Vascular permeability in the retina was quantitated after 3–4 weeks of diabetes using an Evans blue quantitation technique as described previously [17]. Briefly 3–4 weeks after the onset of diabetes or 24 hours after intravitreal injections, mice were anesthetized with isofluorane and injected intravenously (via the tail vein) with 200 µl of Evans Blue dye (Sigma) at a concentration of 45 mg/Kg. Quantitation of the retinal barrier breakdown was assessed as described previously [18]. Additionally, retinas from Evans Blue injected mice were examined by fluorescence microscopy (Nikon). For quantitative analysis of vascular leakage, retinal images were batch processed using a customized macro generated in Image-Pro Plus 6.1 (Media Cybernetics, Silver Spring, MD). In each image, leakage areas were segmented using a gray-level threshold set between 230–255. Intensely stained vessels protruding from binarized leakage areas were removed using successive passes of morphological erosion and dilation filters; areas of processed, segmented regions were then exported to Excel. In original, unprocessed images, a threshold value obtained from an automated search for histogram minima was used to segment the entire retina from background for percent leakage calculations (leakage area divided by total retinal tissue area). Finally, boundaries of binarized leakage regions were extracted using a Sobel filter, pseudo-colored, and then superimposed upon original images to allow visualization of segmented vascular leakage regions.

## Results

### Increased soluble betacellulin in the retinas of diabetic mice with hyper-permeable retinal vessels

**Figure 1:**
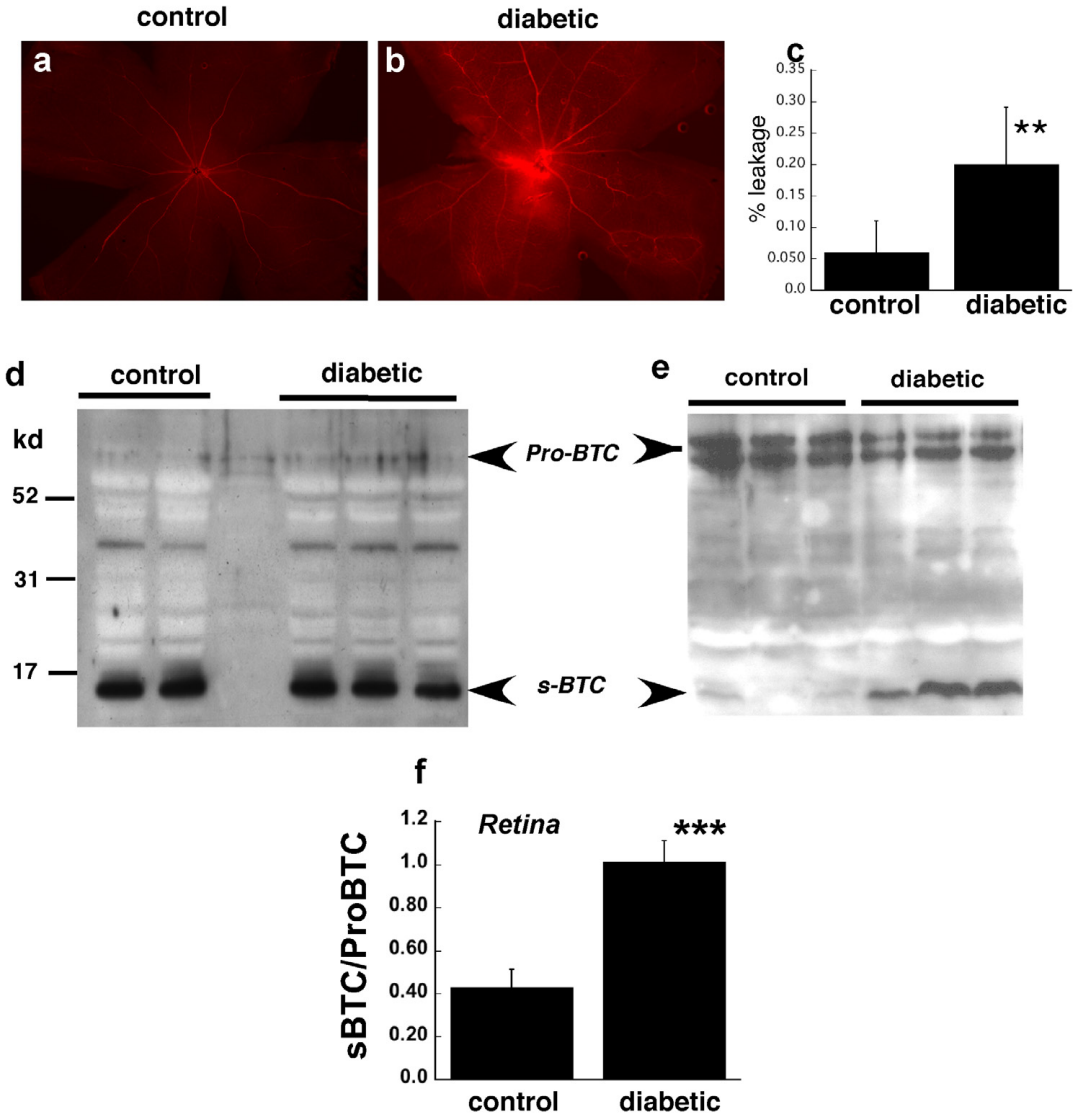
Diabetic mice show augmented retinal vascular permeability and increased expression of cleaved betacellulin in the retina. Leakage of Evans Blue dye from mouse retinal vessels in representative a) normoglycemic and b) hyperglycemic mice was quantitated in whole mount retinas one hr after perfusion with the dye(n = 8). (c). Representative Western blot analysis of betacellulin in pancreas (d) and retinas (e) from control and streptozotocin-induced diabetic mice. (f) densitometric quantitation of the ratio of sBTC to ProBTC in mouse retina from control (n = 12) and diabetic (n = 12) mouse eyes. ***p = 0.008 (ANOVA).

Steptozotocin-induced diabetic mice with hyperglycemia (fasting blood sugar ≥200 mg/ml) were examined for increases in retinal vascular permeability using an Evans Blue leakage assay. As described previously [17], we observed a modest increase in dye leakage from the retinal vessels of diabetic mice (Fig. 1b,c) when compared with control normoglycemic mice (Fig. 1a,c). We examined the expression of betacellulin in the pancreas and retinas of control and diabetic mice. It was interesting to note that in the pancreas the majority of betacellulin was present as the cleaved soluble form (s-BTC) compared with the abundance of the higher molecular weight membrane bound pro-form (Pro-BTC) in normal mouse retinas (Fig. 1e). There appeared to be a trend towards a slight increase in the pro-BTC in the pancreas of diabetic mice (Fig. 1d). In the mouse retina the cleaved low-molecular weight form of active soluble betacellulin (s-BTC) was significantly increased in hyperglycemic mice (Fig. 1e,f) when examined by western blot analysis.

### Increase in retinal soluble betacellulin causes increased retinal vascular permeability in diabetic mice

**Figure 2:**
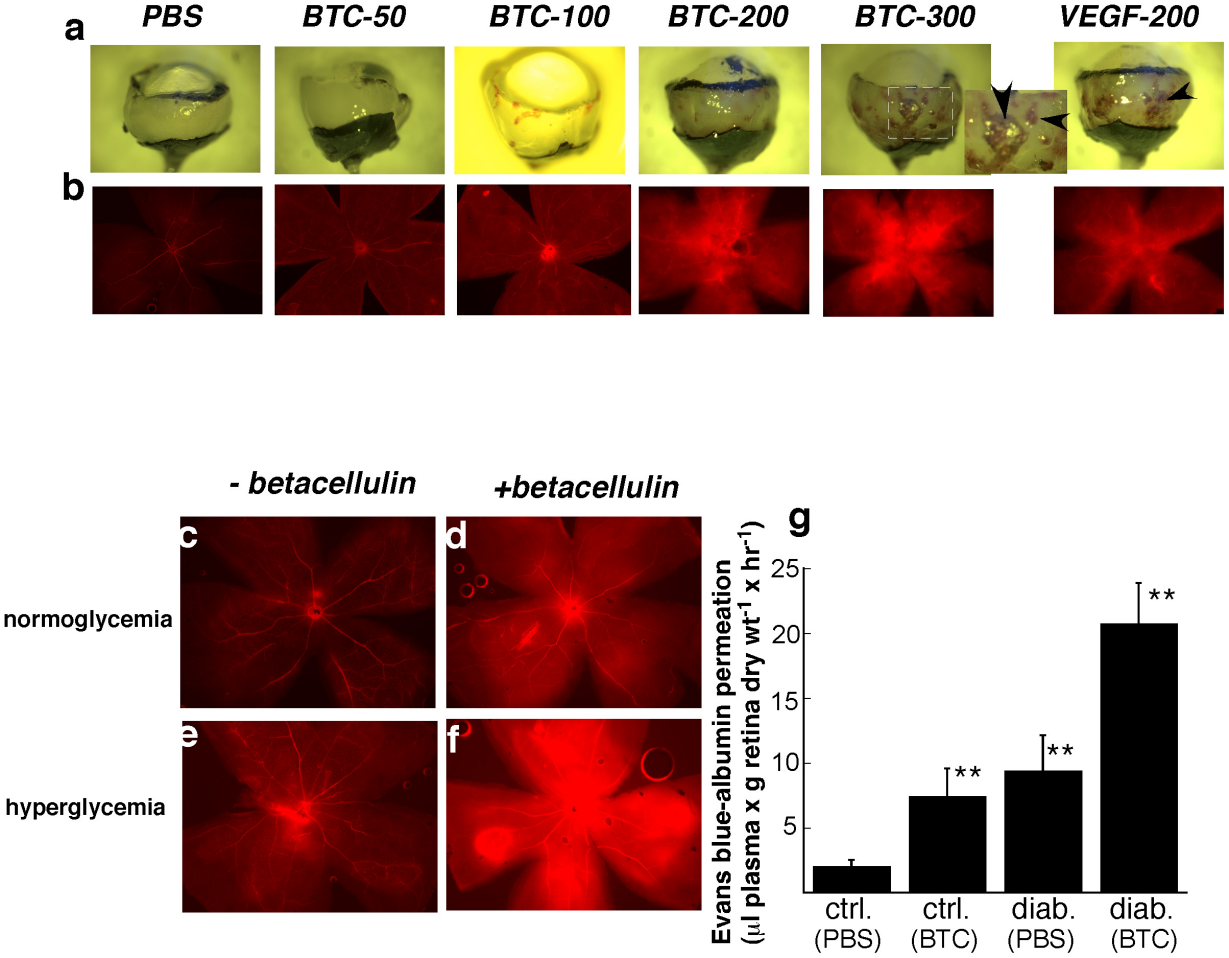
Intravitreal injection of betacellulin induces increased retinal haemorrhage and augmented retinal vascular permeability in normal and diabetic retinas. Dose response of the effect of intravitreal injection of betacellulin (50–300 ng), VEGF (200 ng) and PBS in normoglycemic mice on a) retinal haemorrhage as seen in a bright field image of dissected posterior pole of mouse eyes. Arrowheads indicate areas of hemorrhage. For BTC-300, inset is magnification of boxed area b) Evans Blue dye leakage in flat mounted retinas. Leakage of Evans blue dye from retinal vessels of normoglycemic (c,d) and hyperglycemic (e,f) mice injected intravitreally with PBS(c,e) or 200 ng recombinant mouse betacellulin (d,f). Results are quantitated (g). * p<0.009.

To determine if the increase in soluble active betacellulin seen in diabetic mice could contribute to increased vascular permeability we examined whether increased concentration of betacellulin in the retina could induce retinal vascular leakage in normal mice. Intravitreal injections of increasing doses of recombinant soluble betacellulin (0–300 ng) resulted in increased retinal hemorrhage (Fig. 2a upper panel,arrowheads) and increased retinal vascular permeability (Fig. 2b lower panel) in a dose dependent manner, similar to that induced by vascular endothelial growth factor (VEGF).

### Combination of hyperglycemia and increased intravitreous betacellulin causes accentuated retinal vascular leakage

Since all patients with diabetes do not develop macular edema, we postulated that a combination of hyperglycemia and increased intravitreous betacellulin might lead to a more severe phenotype. Injection of 200 ng of recombinant betacellulin intravitreally in hyperglycemic mice induced a marked accentuation of retinal vascular leakage as detected by extravasation of intravascular Evans Blue dye (Figs. 2f,g) when compared with leakage induced in normoglycemic mice (Figs. 2d,g).

Since VEGF has been proposed to be a critical factor in the increase in retinal vascular permeability in proliferative diabetic retinopathy (PDR) and diabetic macular edema (DME), we wanted to determine if the effect of betacellulin was VEGF-dependent. No increase in VEGF was observed in mouse retinas injected with betacellulin (Fig. S1) indicating that betacellulin increased retinal vascular permeability via a VEGF-independent pathway.

### Increased expression of soluble betacellulin in human diabetic retinas

**Figure 3:**
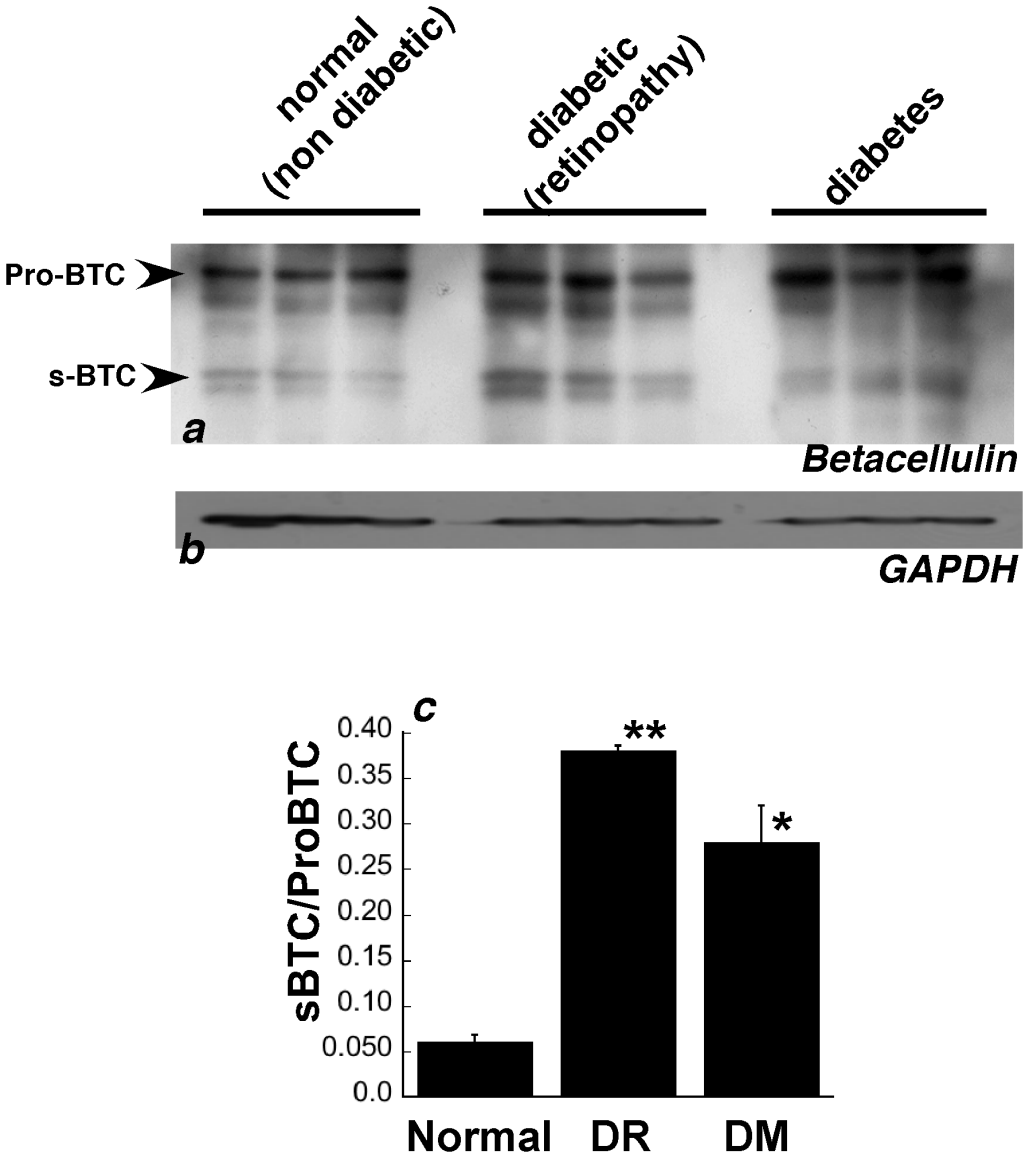
Increased expression of betacellulin in retinas of humans with diabetes. Western blot analysis of (a) BTC (b) GAPDH in retina tissue from human donors with no diabetes, diabetes or diabetes with diabetic retinopathy. (c) Densitometric quantitation of the ration of sBTC to full length BTC using GAPDH as a reference. ** p = 0.01 * p = 0.03.

Betacellulin is expressed as a membrane anchored precursor protein (pro-betacellulin) containing an extracellular N-terminal ectodomain, a transmembrane domain and a cytoplasmic domain[13], [19]. Like other members of the EGF family, the ectodomain of betacellulin can be proteolytically cleaved to release a soluble form of the protein. Protein levels of the pro-betacellulin as well as its cleaved soluble form were examined by western blot analysis in post-mortem retina tissue from healthy donors, patients with diabetes with no clinical diagnoses of retinopathy and patients with diabetic retinopathy. Diabetic retinas showed evidence of increased pro-BTC (38 kD) as well as the cleaved s-BTC (19 kD) compared with retinas from controls (Fig. 3a). Both the membrane anchored and soluble forms of betacellulin are believed to be biologically active, binding to ErbB receptors. The ratio of s-BTC to pro-BTC was significantly increased in the retinas of patients with diabetes (Fig. 3c).

### Increased expression of ADAM10 in diabetic mouse and human retinas

**Figure 4:**
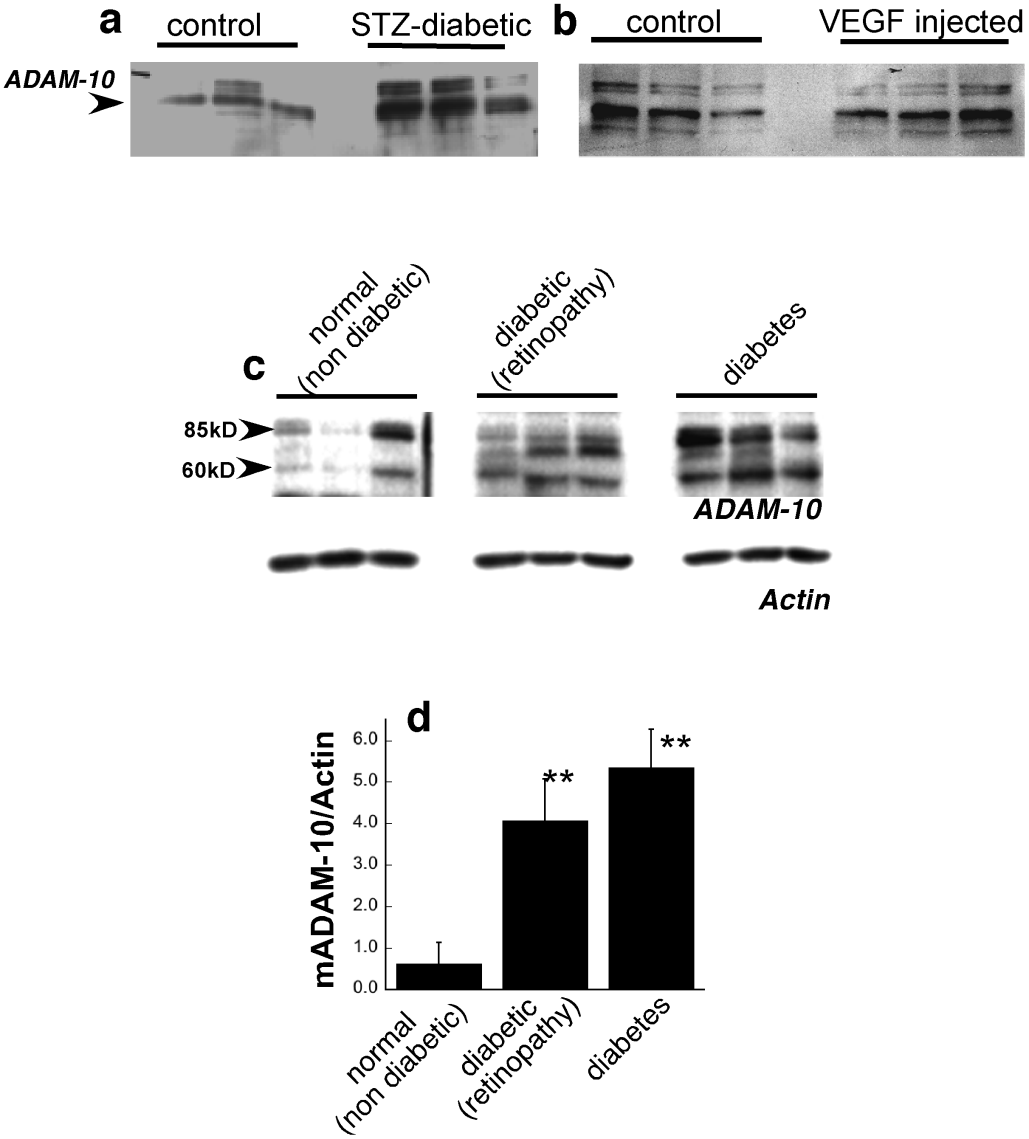
Increased expression of ADAM-10 in diabetic retinas. Western blot analysis of ADAM-10 in retina tissue from (a) control and STZ induced diabetic mice (b) control and intravitreal VEGF injected mice and (c) human retinas from patients with no diabetes, diabetes or diabetes with diabetic retinopathy(d) Densitometric quantitation of mature ADAM-10 (mADAM-10) relative to actin on the same blot. ** p≤0.03.

Recent studies have determined that ADAM-10 (a disintegrin and metalloproteinase-10) can mediate the constitutive and activated shedding of pro-betacellulin[20], [21]. In order to determine if ADAM-10 plays a role in the increased expression of the soluble form of betacellulin in the retinas of diabetic mice we performed western blot analyses using an ADAM-10 antibody. We found that ADAM-10 (62 kD mature form) was increased in the retinas of streptozotocin-induced hyperglycemic mice (Fig. 4a) but not in mice injected intravitreally with recombinant VEGF (Fig. 4b). Immunoblot analysis on retinas from diabetic patients with without a clinical diagnosis of retinopathy determined an increase in mature ADAM 10 when compared with retinas from healthy controls (Fig. 4c,d). While the exact mechanism by which ADAM-10 is increased in the retinas of diabetics remains to be determined, these results indicate that ADAM-10 may contribute to the cleavage of membrane bound betacellulin that causes increased retinal vascular permeability seen as a complication of the disease.

## Discussion

Diabetes mellitus is a global disease with considerable morbidity and mortality. The total number of people with diabetes is expected to reach 300 million by the year 2025, with the majority of increased cases predicted to be in developing countries. The disease is classified into type I diabetes (previously referred to as “insulin dependent” or “juvenile onset”), characterized by an absolute insulin deficiency as a consequence of destruction of the insulin secretory pancreatic β cells of the islets of Langerhans; and type II diabetes (“non-insulin dependent” adult-onset” which is characterized by an insulin secretory defect of the β-cell and insulin resistance in peripheral tissues. Diabetic retinopathy is a common complication of both type I (50% incidence) and type II (30% incidence)[22], [23], [24], [25], [26] diabetes. Diabetic retinopathy can be broadly divided into two clinical stages: non-proliferative and proliferative diabetic retinopathy (PDR). In non-proliferative diabetic retinopathy, the earliest visible sign is the formation of microaneurysms. Increased capillary permeability, results in the leaking of fluid into the surrounding retinal tissue, pooling around the macula causing macular edema and loss of visual acuity. It is believed that the retinal neovascularization in PDR results from ischemia and the consequent release of local hypoxic and pro-angiogenic factors such as VEGF[27], hypoxia-inducible factors[28] and erythropoietin[29]. These factors may stimulate abnormal vessel growth extending into the vitreous of the eye, leading to hemorrhage and visual loss. Laser photocoagulation as well as anti-VEGF therapies have shown significant promise in the treatment of proliferating vessels in PDR. While PDR appears to be extremely responsive to treatment with VEGF inhibitors, early studies indicate that the response to these drugs in retinal edema is often partial and requires higher doses[30]. However, DME appears to be more resistant to these treatment approaches, suggesting that other factors might contribute to the retinal vascular leakage. These results and other recent studies have suggested that VEGF-independent mechanism(s) such as carbonic anhydrase[31], [32] and erythropoietin[33], [34], play a role in diabetic retinal edema. Our results suggest that cleaved betacellulin contributes to increased retinal vascular leakage in diabetes.

ADAMs and ADAM-10 in particular have been recently reported to be novel regulators of vascular permeability[7], [35], [36] and our data indicates that this might occur via increased expression of betacellulin. Cleavage of the membrane-anchored forms of BTC to release a secreted form occurs principally by ADAM-10 (a disintegrin and metalloprotease-10)[20], [21], [37], [38] as well as endothelin-1[39]. ADAM-10 has recently been shown to induce endothelial cell permeability via regulation of VE-cadherin-dependent endothelial cell functions[36]. Whether cleavage of betacellulin is necessary for the increased vascular permeability by ADAM-10 occurs in the retina or elsewhere remains to be determined.

We have examined betacellulin levels in the plasma and found no significant increase in betacellulin in the circulating plasma of patients with PDR (n = 23) (Fig. S2). While this suggests that the betacellulin might be synthesized or processed in the retina we cannot exclude the possibility that the ELISA used to measure betacellulin may lack the sensitivity to detect small changes in circulating levels.

Recent studies have suggested *Neurod-Btc* gene therapy to be a promising approach to induce islet regeneration for the treatment of insulin-dependent diabetes[40]. Our data suggest that we should proceed with caution in this regard as there is a likelihood of inducing or worsening the ocular complications of macular edema. We observed that the magnitude of the effect of intravitreal betacellulin on retinal vascular permeability was equivalent to that of VEGF. While much attention has been focused on the role of VEGF in the pathophysiology of PDR and DME, it is becoming increasingly evident that other vasopermeability factors are involved [32], [41] and more attention should be focused on targeting these factors as therapeutics. Recent findings have also suggested the need to re-evaluate the current diagnostic criteria for diabetes, and the risk of complications based on fasting plasma glucose levels, as neither macrovascular nor microvascular complications seem to respect a strict glycemic threshold [3]. Perhaps glycemia in combination with additional vasopermeability factors might be a more reliable prognostic indicator. In summary, our data suggests that betacellulin plays an important role in the development of increased retinal vascular permeability in diabetes and inhibitors of ADAM-10 or betacellulin signaling might be clinically useful in preventing the development and progression of macular edema in this disease.

## Supporting Information

Figure S1 Betacellulin induces retinal vascular leakage independent of VEGF. Retinas from mice injected intravitreally with PBS or betacellulin (200 ng) or no injection (control) were evaluated for VEGF protein 24 hours post injection.
(0.74 MB TIF)

Figure S2 No significant increase in circulating plasma betacellulin in patients with proliferative diabetic retinopathy. Plasma was prepared from whole blood from patients with no diabetes, diabetes with no evidence of retinopathy and proliferative diabetic retinopathy and analyzed by ELISA for betacellulin.
(0.54 MB TIF)
