# Supplementary figures and images for: Betacellulin Induces Increased Retinal Vascular Permeability in Mice
Source: PLoS One. 2010 Oct 18;5(10):e13444. doi: 10.1371/journal.pone.0013444 (PMC2956654; doi:10.1371/journal.pone.0013444)

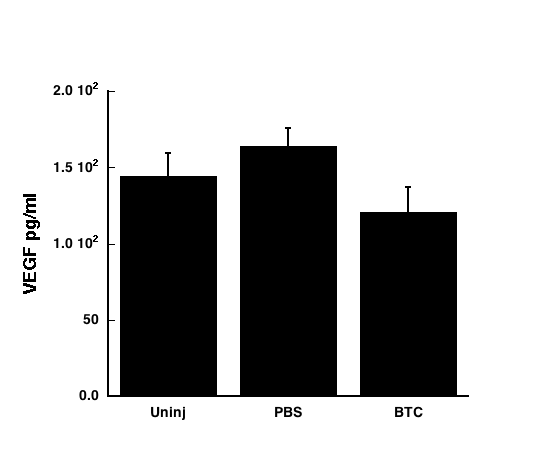

Supplement: Figure S1 — Betacellulin induces retinal vascular leakage independent of VEGF. Retinas from mice injected intravitreally with PBS or betacellulin (200 ng) or no injection (control) were evaluated for VEGF protein 24 hours post injection. (0.74 MB TIF) [file pone.0013444.s001.tif]
